# Non-destructive monitoring of netted muskmelon quality based on its external phenotype using Random Forest

**DOI:** 10.1371/journal.pone.0221259

**Published:** 2019-08-19

**Authors:** Liu Qian, Li Daren, Niu Qingliang, Huang Danfeng, Chang Liying

**Affiliations:** School of Agriculture and Biology, Shanghai Jiao Tong University, Shanghai, People's Republic of China; Fred Hutchinson Cancer Research Center, UNITED STATES

## Abstract

The internal phenotypes of netted muskmelon (*Cucumis melo* L. *var*. *eticulates* Naud.) are always associated with its external phenotypes. In this study, the parameters of external phenotypic traits were extracted from muskmelon images captured by machine vision, and the internal phenotypes of interest to us were measured. Pearson analysis showed that most external phenotypic traits were highly correlated with these internal phenotypes in muskmelon fruit. In this study, we used the random forest algorithm to predict muskmelon fruit internal phenotypes based on the significantly associated external parameters. Carotenoids, sucrose, and total soluble solid (TSS) were the three most accurately monitored internal phenotypes with prediction *R*-squared (*R*^2^) values of 0.947 (root-mean-square error (RMSE) = 0.019 mg/100 g), 0.918 (RMSE = 3.233 mg/g), and 0.916 (RMSE = 1.089%), respectively. Further, a simplified model was constructed and validated based on the top 10 external phenotypic parameters associated with each internal phenotype, and these parameters were filtered with the varImp function from the random forest package. The top 10 external phenotypic parameters correlated with each internal phenotype used in the simplified model were not identical. The results showed that the simplified models also accurately monitored the melon internal phenotypes, despite that the predicted *R*^2^ values decreased 0.3% to 7.9% compared with the original models. This study improved the efficiency and accuracy of real-time fruit quality monitoring for greenhouse muskmelon.

## Introduction

Netted muskmelon (*Cucumis melo* L. *var*. *eticulates* Naud.) is a widely grown fruit in China. Its external and internal phenotypes, such as surface netting, color and nutrient contents, are easily affected by environmental factors. With increasing demands from consumers and the market, it is necessary for growers to monitor muskmelon internal phenotypes without fruit destruction. Previous studies [1, 2] have shown that the muskmelon external phenotypes (i.e., fruit color and skin netting) and internal phenotypes are closely related. Thus, the non-destructive testing of fruit internal phenotypes can be achieved through monitoring fruit appearance, namely, through fruit external phenotypes. Several techniques have been employed to assess internal phenotypes and maturity of muskmelon fruits; these techniques include electronic nose, specific gravity, near infrared spectroscopy (NIRS), chlorophyll fluorescence and machine vision [3].

Due to the ease-of-use, low cost and high adaptability, machine vision has been widely used for image acquisition [4]. Machine vision technology had been used in monitoring parameters relevant to plant biomass [5], yield [6], soil nutrition, hydration [7, 8], product quality [9, 10], germination, etc. Although real-time non-destructive testing technology has improved experimental efficiency, it is still confronted with certain problems in processing large amounts of data. Fortunately, random forest classification performs well on this type of problem [11].

According to Wikipedia: “Random forests or random decision forests are an ensemble learning method for classification, regression and other tasks that operates by constructing a multitude of decision trees at training time and outputting the class that is the mode of the classes (classification) or mean prediction (regression) of the individual trees.” random forest. At present, random forest regression and classification have been widely used in estimating yield [12, 13], chlorophyll content [14], leaf nitrogen concentration [15], leaf area index of plant canopy [16], and gene expression [17], etc. Moreover, random forest is more efficient and has advantages over other real-time non-destructive testing technologies.

In this study, ‘wanglu’, a relatively new variety grown in China, was selected as our research subject. A total of 402 ‘wanglu’ fruit images were collected by a machine vision system, and 65 external phenotypic parameters were extracted from each image. Correspondingly, the fruit internal phenotypes, including the contents of sugar, total soluble solid (TSS), vitamin C, chlorophyll *a*, chlorophyll *b*, and carotenoids, were measured destructively [17]. For these internal phenotypes, sugar and vitamin C contents were measured by high-performance liquid chromatography, and pigment was measured by spectrophotometry. Fruits rich in sugar and TSS tend to have high vitamin C content, higher nutritional value, and a flavor profile preferred by most consumers. The pigment content of fruit peel affects its appearance. In this study, 402 samples were randomly divided into three groups. Two third of these samples were used for model construction, and the remaining samples were used for model validation. We then constructed the muskmelon internal phenotypes monitoring models by the random forest algorithm based on muskmelon external phenotypic parameters. The fruit internal phenotypes real-time monitoring system established in this study will reduce the amount of destructive muskmelon sample in determining fruit maturity and harvest time, and it can be applied in fruit automatic grading and then improving fruit distribution efficiency.

## 1. Materials and methods

### 1.1 Materials and measurement methods of fruit internal traits

The experiment was conducted in a greenhouse (32 m×16 m, Venlo-Type, Shanghai Dushi Green Company, China) at Shanghai Jiaotong University (31°11´N, 121°29´W), China. The greenhouse was divided into 16 rows in the east-west direction and each row had 102 pots with 1 plant per pot. There were 102 muskmelon plants in each experimental season. The muskmelons were vertically planted in pots with a total volume of 16 L of substrate containing 2:2:2:1 mixture of meteorite: perlite: peat: organic fertilizer. The bulk density and saturated water content were 0.21 g/mL and 140%, respectively.

Cultivation management was as follows: seedlings were transplanted into pots at the two true leaf stage pruned to a single branch. Planting density was 6.5 plants per square metre. The top growing point was removed when the leaf number of a plant reached 24 and one fruit was set after hand-pollination. The heating, ventilation, internal and external shading of the greenhouse were controlled automatically by the greenhouse computer control system. We set 4 different irrigation treatments, and each treatment was repeated 4 times. The 4 water treatments were alternating 50~60% relative water content (RWC), 60~70% RWC, 70~80% RWC, and 80~90% RWC. All the treatments applied the same irrigation amount of 50–60% for the seedling stage and the same irrigation amount of 55–70% for the extension stage. The plant irrigation management was controlled by an automatic drip system.

A total of 134 fruits of “wanglu”, a cultivar of netted muskmelon (*C*. *melo* L. *var*. *eticulates* Naud.), were produced in either the autumn of 2016 or the spring of 2017 under different substrate water status. The samples were collected every 5 days and continued from the ten days after pollination until fruit matured. The internal phenotypes were determined destructively after being imaged with a machine vision system. Nine fruit internal phenotypes, including the content of fructose, glucose, sucrose, total soluble solid (TSS), vitamin C (Vc), chlorophyll *a*, chlorophyll *b*, carotenoids, were measured as previously reported [17].

### 1.2 Machine vision system and sample image

**Fig 1:**
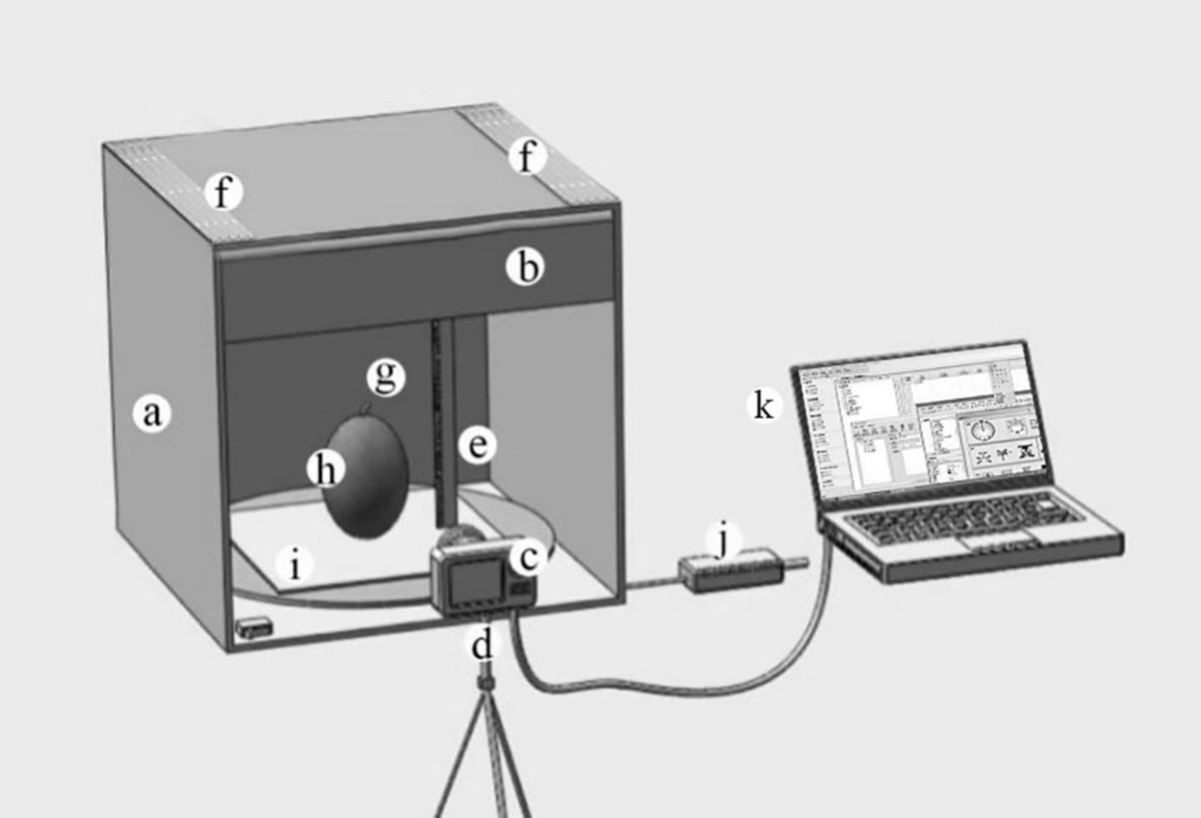
Machine vision system. a, photo box (60 cm×60 cm); b, curtain; c, single lens reflex (SLR) camera (Canon, Japan); d, camera tripod; e, straightedge; f, aluminum (Al) panel; g, specialized backdrop; h, muskmelon; i, shadowless lamp (30 cm×30 cm); j, 10-gear dimmable driver; k, computer.).

The machine vision acquisition system shown in Fig 1 was built by our research team. It has stable intensity illumination, which is necessary for consistent imaging. The parameters of the single lens reflex (SLR) camera (Canon ES05, Japan) settings were as follows:M-gear, shuttle speed = 1/320 s, focal length = 60 mm, exposure compensation value = 0, and ISO = 200. In addition, a total of 60 light emitting diode (LED) lamps (the highest power was 60 W) controlled by a 10-gear dimmable driver, were installed on the two aluminium (Al) panels fixed on two-sides of a photo box. The photo box can be opened from the top and two sides.

After fruiting, a total of 130 muskmelon fruit samples were collected from different planting seasons and taken back to lab or 10 samples every five days. Each sample was imaged with machine vision before measuring internal phenotypes. The sample was placed on the centre of a shadow-less lamp equipped with a rotating base, and pictures were taken at 35-cm distance from three angles with 120° interval from each other, and color calibration cards (RAL-K7, Germany) and transparency scale plate was used as correction standard for color and size, respectively. The fruit image in 5616*3744 pixels was outputted in JPG format and stored in a secure digital memory (SD) card, which can be exported from USB interface.

### 1.3 Image processing step

**Fig 2:**
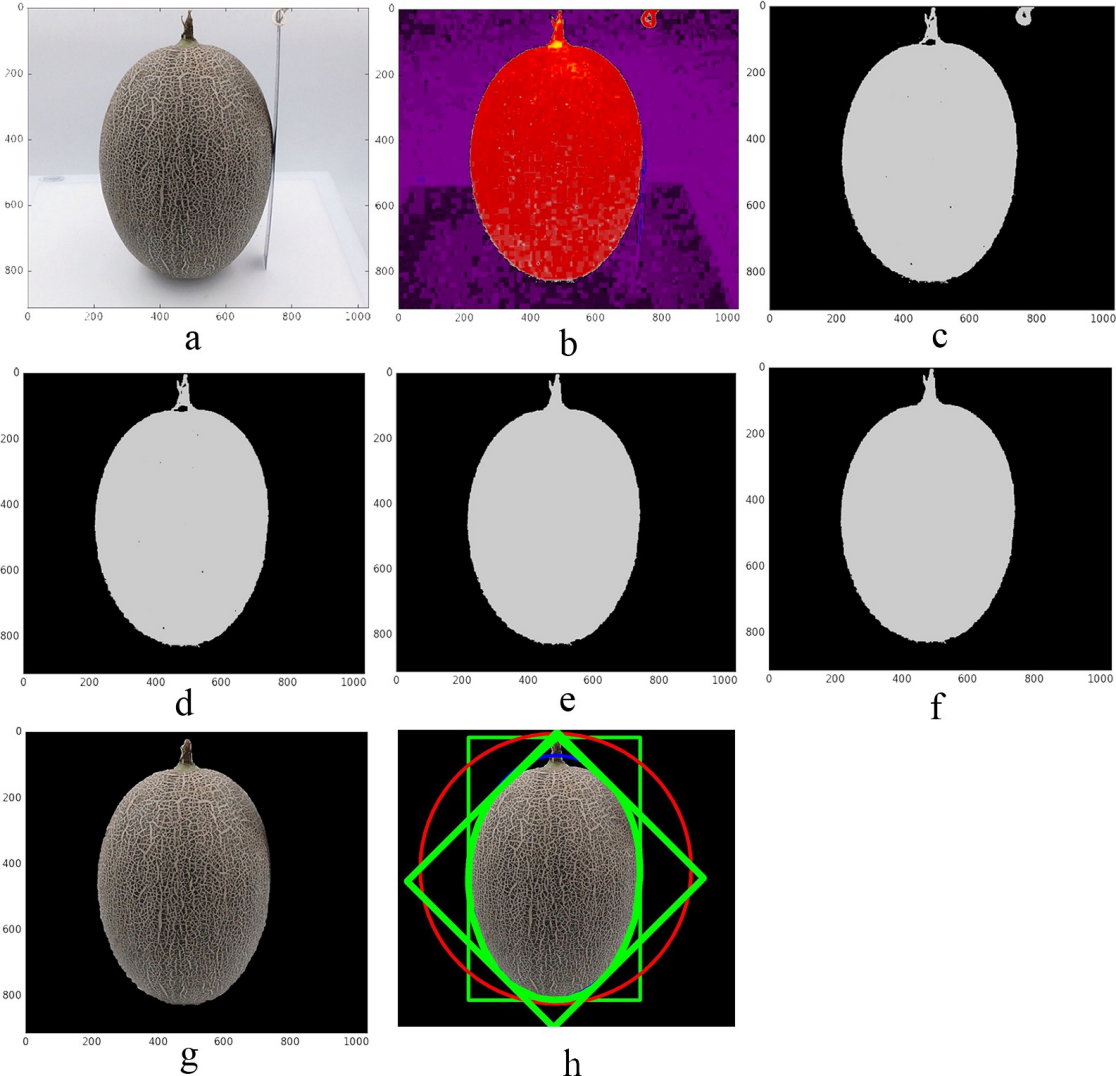
Muskmelon image processing. a: original image; b: original image is processed with 2B-R-G (deformation of the super-green's method—2G-R-B in muskmelon); c: threshold filtered image mask for b-image; d: remove small objects from c-image; e: remove small holes from d-image; f: open operation for e-image; g: segmented image obtained after f-image masking; h: morphological parameters extraction sketch.

To ensure forecasting result accuracy, all of the extracted external phenotypic parameters were matched to certain internal phenotypes. The image processing method was as follows (Fig 2): i) filtering original image background by Open-CV (Open-CV Foundation, USA); ii) correcting image characteristics with an image-like enhancement algorithm to avoid color and netting distortion; and iii) maximizing image preservation. For each image, a total of 65 parameters were extracted, which included 45 color parameters, 6 netting traits’ parameters, and 14 parameters of morphological traits (Table 1). The color parameters were the mean, SD, median, range, and coefficient of variation (CV) of each of the nine color traits (red (R), green (G), blue (B), lightness (L), color channel a, color channel b, hue (H), saturation (S), value (V)) involved in three color space models, RGB, lab, and HSV. The six netting traits’ parameters were contrast, dissimilarity, homogeneity, energy, correlation, and angular second moment (ASM).

**Table 1:** Parameters of muskmelon color, netting, and morphology traits.

No.	Extracted Index	Description	Category
1–5	R mean/SD/median/Range/CV	Red	Color
6–10	G mean/SD/median/Range/CV	Green	
11–15	B mean/SD/median/Range/CV	Blue	
16–20	L mean/SD/median/Range/CV	Brightness	
21–25	a mean/SD/median/Range/CV	Color channel	
26–30	b mean/SD/median/Range/CV	Color channel	
31–35	H mean/SD/median/Range/CV	Hue	
36–40	S mean/SD/median/Range/CV	Saturation	
41–45	V mean/SD/median/Range/CV	Value	
46	Contrast	Definition and grooving depth of texture	Texture
47	Dissimilarity	The difference of grey scale	
48	Homogeneity	The local changes of image texture	
49	Energy	Degree of thickness and uniformity of texture	
50	Correlation	The correlation of local grey scale	
51	ASM	Angular Second Moment	
52	Contour area	Melon area	Morphology
53	Perimeter	Melon circumference	
54	w	External rectangle width	
55	h	Externally Rectangular High	
56	Hull area	External convex hull area	
57	X-w	The width of the smallest circumscribed rectangle	
58	X-h	The minimum height of the circumscribed rectangle	
59	MA	Melon fits the long axis of the ellipse	
60	ma	Melon fits the minor axis of the ellipse	
61	r	Melon minimum circumcircle radius	
62	Equivalent diameter	Diameter of the same area circle	
63	Aspect ration	Minimum rectangular aspect ratio	
64	Extend	Melon area ratio to straight rectangular area	
65	Solidity	Melon area with convex hull area ratio	

### 1.4 Random Forest model construction and validation

In total, 26,130 parameters (402 images×65 parameters per image) were obtained. In the study, each image was considered as a single RF model and was trained with ntree as 300 and mtry from 1 to 100 [18]. For the feature selections of the simplified models, we chose the mean squared error to represent the criteria of relative importance in RF models. Moreover, we adopted 10-fold cross-validation and a three repetition strategy to check the prediction power of each model. Evaluation parameter for model accuracy included the root-mean-square deviation (RMSE), mean absolute deviation (MAE) and *R*^2^ between forecasted and measured values. A relatively higher *R*^2^ value and lower RMSE value suggested accurate prediction results. They were calculated as:

$$R^{2}=\frac{\sum_{i=1}^{n}{\left({\hat{y}}_{i}-\bar{y}\right)}^{2}}{\sum_{i=1}^{n}{\left(y_{i}-\bar{y}\right)}^{2}}$$

$$RMSE=\sqrt{\frac{1}{n}\sum_{i=1}^{n}(y_{i}{-\hat{y_{i}})}^{2}}$$

where ${\hat{y}}_{i}$ is the estimated fruit internal phenotype value, *y_i_* is the measured value, $\bar{y}$ is the mean of measured value, and *n* is the number of measured value in the validation data.

All the correlation analyses were operated by random forest package in R 3.4.1 software and the R codes were performed on the RStudio-0.98.109 platform.

## 2. Results

### 2.1 Data analysis of fruit internal traits

The fruit internal phenotype parameters are shown in Table 1. The establishment of fruit quality monitoring models based on machine learning requires the support of a large amount of variability and dynamic and variant data. Hence, before model construction, diverse muskmelon samples and many real-time data were collected. A good data set is a prerequisite for establishing robust models.

**Table 2:** Statistics analysis of all melon samples and the performance of original and simplified RF models.

Internal trait	Statistics analysis				Original random forest model				Simplified random forest model			
	Sample no.	Maximum	Minimum	Mean(Std)	Mtry =	RMSE	*R*^2^	Training time (s)	Mtry =	RMSE	*R*^2^	Training time (s)
Fructose (mg/g)	123	71.12	4.79	16.83±3.75	3	5.092	0.761	43.65	1	5.034	0.750	37.55
Glucose (mg/g)	123	72.25	0.21	9.90 ±3.18	5	5.111	0.758	49.94	2	6.553	0.735	43.98
Sucrose (mg/g)	123	46.90	1.05	12.99 ±4.23	11	3.233	0.918	107.69	3	4.447	0.839	38.19
Total sugar (mg/g)	123	164.20	10.20	39.71±10.32	5	11.697	0.821	75.70	1	11.254	0.818	25.90
Total soluble solid (TSS, %)	101	18.50	5.80	10.64±3.20	7	1.089	0.916	72.99	4	1.135	0.908	35.63
Vitamin C (mg/100 g)	129	110.84	3.38	41.11 ±16.75	18	11.637	0.585	162.43	17	10.707	0.529	103.31
Chlorophyll *a* (mg/g)	130	1.64	0.27	0.84 ±0.23	19	0.157	0.681	173.76	19	0.163	0.653	95.08
Chlorophyll *b* (mg/g)	130	1.12	0.16	0.39 ±0.14	27	0.074	0.815	229.36	24	0.071	0.808	100.65
Carotenoids (mg/100 g)	101	0.40	0.12	0.24 ±0.05	9	0.019	0.947	63.10	7	0.021	0.936	52.24

### 2.2 Selecting parameters for model construction

**Fig 3:**
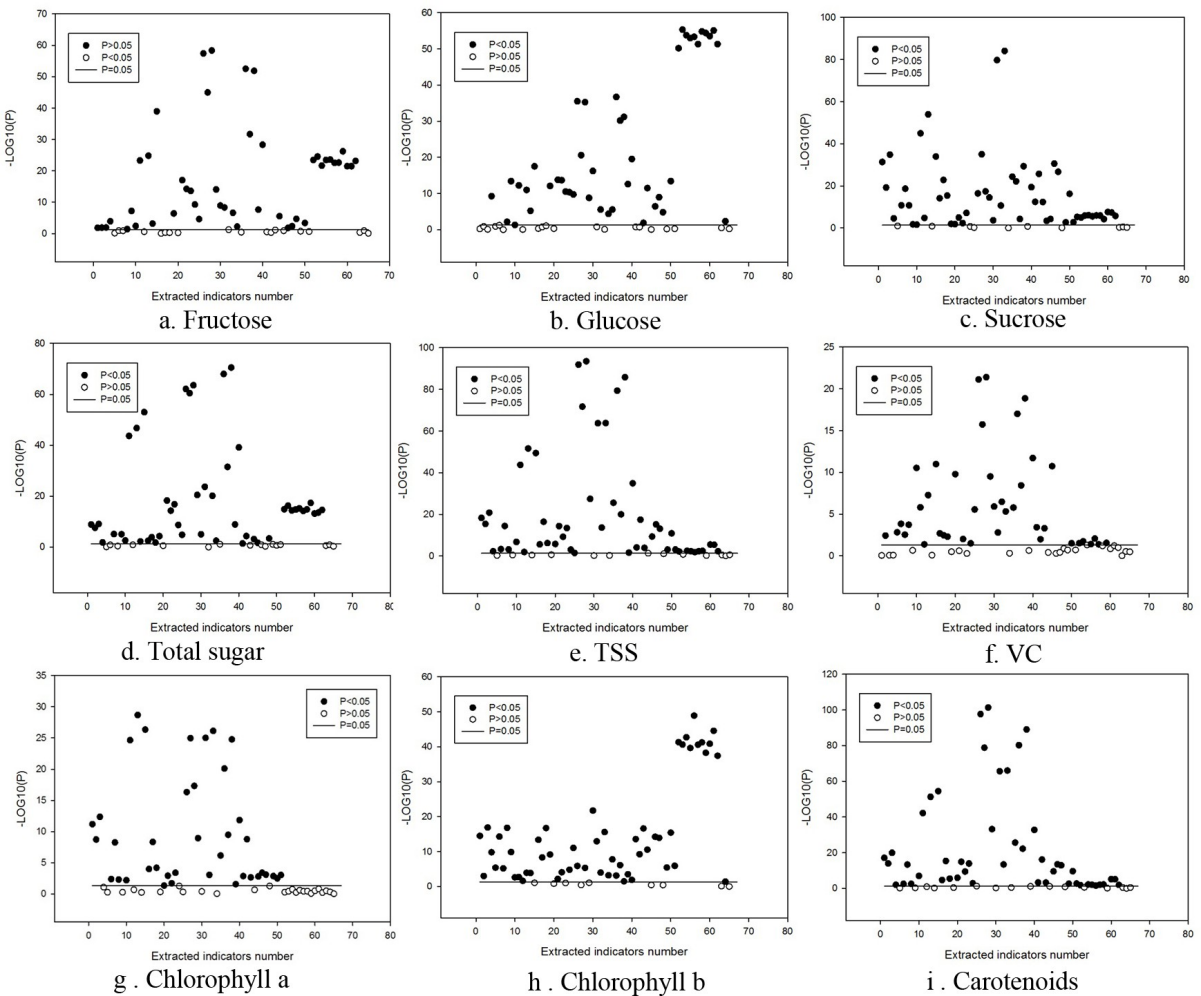
The correlation analysis between internal and external phenotypic parameters of muskmelon. The solid horizontal line represents P = 0.05, i.e.,–log10 P = 1.301. (The abscissa refers to the number of each indicator in Table 1. In order to visually show the correlation, the P value of the ordinate significance test is converted to -LOG10(P).

The relationship of fruit external and internal traits were conducted by Pearson correlation analysis [19–21] (Fig 3) and the external phenotypic parameters significantly associated with internal phenotypes (*P*<0.05) were used for constructing the model (Table 2). Each parameter obeys a normal distribution.

Results showed that most of the external phenotypic parameters were significantly correlated with the internal phenotypes. In addition, the relevant external phenotypic parameters of each internal phenotype were different in both number and type. For example, the chlorophyll *b* content in fruit skin was significantly correlated with 56 external phenotypic parameters, including 39 color parameters, 5 netting parameters, and 12 morphology parameters. Chlorophyll *b* had the most related external phenotypic parameters among all the internal phenotypes.

### 2.3 Random Forest model construction and validation

All the external phenotypic parameters showing significant correlation with internal phenotypes were used for constructing the original forecast model. After validation, the *R*^2^ parameter for evaluating model accuracy were >0.75 for most of the internal phenotypes, except vitamin C and chlorophyll *a* contents in fruit skin, indicating that all the internal phenotypes were well-predicted. Further, the *R*^2^ values for predicting sucrose, TSS, and carotenoid were the highest, which were 0.947 (RMSE = 0.019 mg/100 g), 0.918 (RMSE = 3.233 mg/g), and 0.916 (RMSE = 1.089%), respectively (Table 1).

### 2.4 Analysis of the contribution value of external phenotype parameters to internal phenotypes

**Fig 4:**
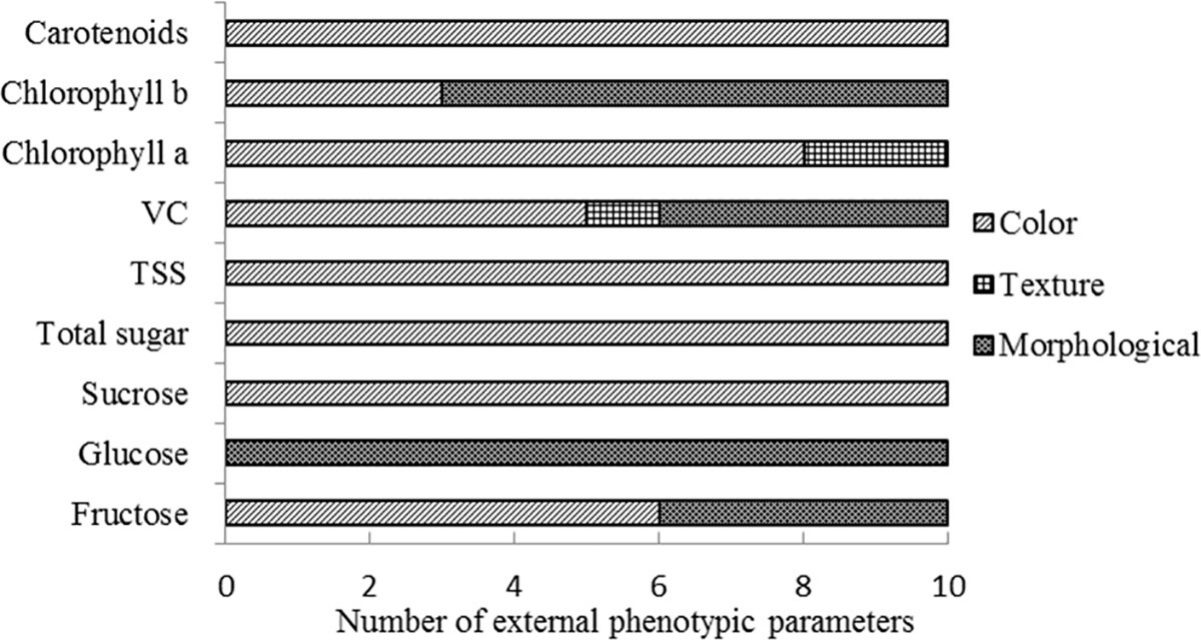
The number of external phenotypic parameters included in the top 10 parameters for each internal trait.

The varImp function in random forest was used to analyse the contribution value of each external parameter to internal phenotypes in the original forecast model. The top 10 parameters associated with each internal phenotypic trait were chosen to construct a simplified forecast model to shorten the model running time (Table 2 and Fig 4).

The top 10 external phenotypic parameters for sucrose and total sugar contents were all color parameters. Among these parameters, H-median had the most contribution to sucrose content, followed by B, R, G and I, whereas S-median was the highest contributor for total sugar content, then followed by b, B, and H. In addition, the top 10 external phenotypic parameters of glucose content were all related with the morphology traits of r, MA, ma, X, h, hull-area, w, perimeter, X-w, h, and equivalent diameter. For fructose, the related top 10 external phenotypic parameters included 6 color parameters from three color spaces and 4 morphology parameters (ma, hull-area, h, and r). These results indicated that color space and color component effects fruit sugar content.

The top 10 external phenotypic parameters for TSS were all color parameters, including median, SD, and mean in trait b of Lab, median and mean in trait S of HSV, as well as median and B mean in trait H of RGB. However, the top 10 parameters of Vc comprised parameters correlated with the netting trait, morphology traits (perimeter, MA, h, hull-area), as well as color traits (S median, S mean, b median, b mean, H median), suggesting the complexity of predicting Vc compared with other internal quality traits.

As for the top 10 external phenotypic parameters for three kinds of pigments in fruit skin, 7 morphology and 3 color parameters were associated with chlorophyll *b*; 8 color and 2 netting parameters were associated with chlorophyll *a*, and 10 color parameters were exclusively associated with carotenoids.

### 2.5 Simplified random forest model with top 10 phenotypic parameters and validation

A simplified random forest model was established based on the top 10 external phenotypic parameters related with each internal phenotype. After validation, all the *R*^2^ values for forecasting internal phenotypes with simplified random forest model were more than 0.75, and among them, the *R*^2^ of forecasting carotenoids and TSS contents were 0.936 (RMSE = 0.019 mg/100 g) and 0.908 (RMSE = 1.135%), respectively, suggesting that the simplified model forecasted the internal phenotypes well (Table 2).

## 3. Discussion

In the study, with machine vision technology, the external phenotypic parameters of muskmelon samples from different planting seasons, different growth stages, and under different substrate water status were extracted and used for constructing a simplified random forest model. When forecasting muskmelon internal phenotype with this model, the *R*^2^ values for 7 of 9 internal phenotypes were >0.75, indicating that muskmelon external phenotypes were highly associated with their internal phenotypes, which is similar to the results of Wang et al. [9] and Wei et al. [22]. Moreover, the *R*^2^ values for sucrose, carotenoids, and TSS were 0.918 (RMSE = 3.233 mg/g), 0.947 (RMSE = 0.019 mg/100 g), and 0.916 (RMSE = 1.089%), respectively, indicating that the forecast accuracy was improved greatly.

Further, to improve model forecasting efficiency [19], the 10 external phenotypes that contributed most to each internal phenotype in the original model were selected. They were not identical for each trait. The contents of sucrose, total sugar, carotenoids, and TSS were highly associated with color parameters of external phenotypes. Among all the internal phenotypes, sucrose content was a key factor related with muskmelon sweetness which can affect the market value of muskmelon [20]. At present, muskmelon fruit sucrose content can be monitored from its external phenotypes by forecast modelling during fruit development, and prediction accuracy increased with the increase in parameter number in the forecast model. The other important factor related to the fruit taste of muskmelon was TSS [21], a mixture of many compounds such as saccharin acid. It is associated with phenotypic color parameters and could be well forecasted (SD±1.135%). Additionally, the carotenoid content in fruit skin was also associated with the external phenotypic color parameters, which was nearly the same as those for TSS. The same result was also reported by Tao et al. [23].

In addition, among the top 10 contribution parameters of each internal phenotype, the color parameters accounted for the most percentage compared with netting and morphology parameters. As all color parameters were involved in predicting internal phenotype, it is necessary to analyse all color parameters except for the mean.

After simplification, the original and simplified random forest models showed similar forecasting accuracy with *R*^2^ within 0.3–7.9% of what was reported in Guo et al. [18], Svetnik et al. [24] and Heung et al. [25]. The forecast results were similar between the two models because the random forest algorithm was not sensitive to the parameters that were not significantly related with the forecast traits. Thus, the forecast accuracy did not decrease after the model was simplified. That is, the simplified model still satisfied the need of forecasting muskmelon internal traits.

In this study, the muskmelon image was captured under fixed illumination conditions, which are not the same as real production conditions. In real production conditions, the natural light is unstable and may disturb the image effects. Therefore, the random forest model presented here may have problems in real muskmelon production conditions, and further studies are needed.

## 4. Conclusion

The non-destructive estimation of muskmelon fruit internal phenotypes is achievable by the random forest model based on the external phenotypic images captured with machine vision technology. Both the original and simplified models effectively forecasted fruit internal phenotypes, with *R*^2^ value >0.70 for most of estimated internal phenotypic traits (except vitamin C). Moreover, the model can be used for muskmelons at different growth stages and under different substrate water statuses. This study provides new insights into the monitoring of muskmelon internal phenotypes.
